# Developments in the study of the role of lactate metabolism in the genesis and progression of thyroid cancer

**DOI:** 10.3389/fcell.2025.1640454

**Published:** 2025-08-22

**Authors:** Lei Shi, Xuyang Zhong, Jiafeng Liu, Yong Ying, Zibing Liao, Jin Liu, Liwen Zhao, Jianing Zhong, Xiangtai Zeng

**Affiliations:** ^1^ Department of Thyroid and Hernia Surgery, First Affiliated Hospital of Gannan Medical University, Ganzhou, Jiangxi, China; ^2^ Key Laboratory of Prevention and Treatment of Cardiovascular and Cerebrovascular Diseases of Ministry of Education, Gannan Medical University, Ganzhou, China; ^3^ Ganzhou Key Laboratory of Thyroid Cancer, First Affiliated Hospital of Gannan Medical University, Ganzhou, Jiangxi, China; ^4^ Department of Clinical Medicine, First Clinical College, Gannan Medical University, Ganzhou, Jiangxi, China; ^5^ Basic Clinical College, Gannan Medical University, Ganzhou, Jiangxi, China; ^6^ Department of General Surgery, Longnan Hospital, First Affiliated Hospital of Gannan Medical University, Ganzhou, Jiangxi, China

**Keywords:** thyroid cancer, glycolysis, lactate, lactylation, mechanism

## Abstract

Thyroid carcinoma is among the most common endocrine system malignancies. Lactate metabolism and lactylation modification roles in carcinogenesis and development have garnered more interest in recent years. The expression and function of lactate transporters (MCTs) and significant metabolic enzymes are included in our summary of the characterisation of lactate metabolism in thyroid cancer. We systematically dissect the multifaceted regulatory circuits governing lactate metabolism by integrating three pivotal dimensions: (i) canonical signaling cascades; (ii) tumor-microenvironmental variables, most notably hypoxia and acidosis; and (iii) the emerging epigenetic paradigm of lactylation, which dynamically reprograms the catalytic efficiency of rate-limiting enzymes and reshapes the transcriptional landscape of metabolic genes, thereby precisely coordinating lactate homeostasis. Furthermore, this review explains how lactate promotes thyroid cancer cell growth, invasion, metastasis, angiogenesis, and immunosuppression. It also discusses how lactate may contribute to treatment resistance. This paper provides new ideas for future research and clinical translation by summarizing the key findings and clinical significance of the current research on lactate metabolism in thyroid cancer, anticipating future research directions, and applying the development of more effective treatments that target lactate metabolism to clinical practice.

## 1 Introduction

Thyroid cancer has become more common worldwide in recent years, which may be due to environmental factors and advancements in diagnostic technology. Compared to men, young and middle-aged women are far more likely to develop thyroid cancer, and it also affects children and the elderly. Additionally, certain thyroid cancers, particularly medullary carcinoma, show a clear familial pattern (Huang et al., 2024). Thyroid carcinoma comes in four primary forms: differentiated thyroid cancer (most frequently papillary thyroid carcinoma), medullary thyroid carcinoma, undifferentiated carcinoma, and other rare types. When creating treatment regimens, it is crucial to comprehend the molecular mechanisms underlying the evolution of thyroid cancer. Different types of thyroid cancer have varying prognoses and responses to treatment. Even after curative-intent surgery, a subset of patients still experience disease relapse. Moreover, both radioactive iodine therapy and molecularly targeted agents carry unique toxicity profiles, necessitating meticulous risk–benefit deliberations to balance efficacy with safety (Instrum et al., 2025).

In addition to being a crucial energy source, lactate, which is created during glycolysis, also helps keep bodily fluids’ acid-base balance. Studies show that lactate exerts multiple tumour-supporting effects within the micro-environment: it dampens anti-cancer immune responses, stimulates the formation of new blood vessels, and drives extensive remodelling of the extracellular matrix (Kim E. et al., 2025). Thus, focusing on lactate metabolism or associated pathways could develop into a novel tumor treatment approach.

Further molecular mechanism study is required to examine the interaction of lactate with other metabolic pathways, signaling pathways, and cellular activities, as the precise mechanism of lactate in the thyroid gland is poorly understood. Clarifying the regulatory mechanisms of lactate metabolism in thyroid cancer, outlining the role of lactate in the development and metastasis of thyroid tumors, and exploring the potential of lactate and lactylation alterations as a novel therapeutic method for thyroid cancer are the objectives of this review.

## 2 Features of thyroid cancer cells’ lactate metabolism

### 2.1 Lactate dehydrogenase (LDH) and its isoforms (LDHA, LDHB)

Lactate and NAD + are produced by the interconversion of pyruvate and NADH, which is catalyzed by the essential glycolytic enzyme LDH (Tang et al., 2022). The tetrameric enzyme LDH comprises two subunits, LDH-A (also called the M subunit) and LDH-B (sometimes called the H subunit). These subunits can be joined to form five distinct isozymes, and the expression levels of these isozymes vary depending on the tissue (Comandatore et al., 2022). Compared to normal thyroid tissues, thyroid cancer tissues have a noticeably greater overall expression level of LDH, according to an increasing amount of research, and it is especially noticeable in undifferentiated carcinomas (Guo et al., 2024). One often used test is immunohistochemistry (IHC) labeling, which shows a substantial increase in LDH expression in tumor cells (Liu F. et al., 2025). The primary subunit of LDH, LDHA, is crucial to the tumor cells’ glycolysis process. The study discovered that the tissues of several thyroid cancer cell lines and patients’ tumors had much higher levels of LDHA expression, which is frequently linked to tumor aggressiveness, the risk of metastases, and a poor prognosis (Zhao et al., 2022). According to cell culture studies, thyroid cancer cell lines have noticeably higher levels of LDH activity than healthy thyroid cells, and an increase in its activity usually accompanies the increased expression of LDHA (Du et al., 2023), which further promotes the production of lactate to provide energy for tumor cells, while thyroid cancer cell proliferation and metastasis can be considerably reduced by inhibiting LDHA activity (Broecker-Preuss et al., 2016). The expression of the LDH gene can be regulated by specific transcription factors, including c-Myc and hypoxia-inducible factor-1α (Jiang et al., 2025), thyroid cancer cells usually exhibit elevated c-Myc and HIF-1α activity, which promotes LDH expression. The expression and activity of LDH and its subforms can be regulated by the aberrantly activated PI3K/Akt, MAPK, and other signaling pathways found in thyroid cancer cells (Lin et al., 2012).

### 2.2 Pyruvate kinase (PKM) and its isoforms (PKM1, PKM2)

PKM, which is expressed by the PKM gene and produces two primary isoforms by alternative splicing, PKM1 and PKM2, is the last step in the glycolysis pathway to make ATP. PKM can catalyze the production of ATP by converting phosphoenolpyruvate to pyruvate (Li et al., 2024a). According to several studies, PKM2 is the predominant isoform in thyroid cancer, and IHC (immunohistochemistry) tests have revealed that tumor cells exhibit significantly higher PKM2 expression than the surrounding normal tissue (Taniguchi et al., 2021), PKM1 is often expressed either low or not at all. Nevertheless, PKM2 has less catalytic activity than PKM1, and it forms monomers and dimers readily. Its activity is controlled by several variables, including: some kinases (like MAPK) can phosphorylate PKM2, which lowers its activity; acetylation modification will also impact PKM2’s oligomerization state and activity; and FBP (fructose 1,6-bisphosphate) can encourage the formation of tetramers in PKM2, which will activate its activity (Guo et al., 2025). PKM2’s low activity promotes the Warburg effect, which enables tumor cells to accumulate an intermediate product of glycolysis for biosynthesis and maintain rapid growth (Li Q. et al., 2025). Multiple proteins physically interact with PKM2, and PKM2 itself orchestrates gene expression and cell-cycle progression while promoting epithelial-to-mesenchymal transition in tumour cells, thereby enhancing their invasive and migratory capacities (Ding et al., 2025). Furthermore, PKM2 controls tumor cell drug resistance, which impacts the sensitivity of chemotherapy and targeted therapeutic drugs (Wang J. et al., 2025). To distinguish between benign and malignant cancers, the expression level of PKM2 can be utilized as an additional diagnostic marker for thyroid cancer. Treatment for thyroid cancer may benefit from focusing on PKM2 metabolism.

### 2.3 Expression of MCT1 and MCT4 in thyroid cancer cells

Monocarboxylic acids (including lactate, pyruvate, and ketones) are transported into and out of cells by MCT, a class of membrane transport proteins, of which MCT1 and MCT4 are the two most well-studied (Zhang et al., 2024a). Primarily responsible for monocarboxylic acid intake, MCT1 is expressed in most cells, has a high affinity, and uses a proton-coupled co-transport mechanism to transfer lactate into cells and protons out of cells, helping to maintain cell pH (Liu H. et al., 2024). It has a low affinity, transports lactate out of cells, lowers intracellular lactate concentrations, and transports protons into cells, causing extracellular acidification. MCT4 is primarily responsible for exporting monocarboxylic acids, typically expressed in cells with active glycolysis. High expression of MCT4 is a feature of many cancers, including some thyroid cancers (Tang F. et al., 2024). Research has indicated that MCT1 might be involved in the way tumor cells use lactate as an energy source to fuel their growth, but MCT4 expression is more important in thyroid cancer cells, particularly those with high glycolytic activity (Adashek et al., 2022). Tumor cells are thought to use this high expression as an adaptive method to maintain intracellular pH and release stored lactate. The tumor microenvironment frequently contains hypoxia, which can trigger the expression of HIF-1α, which can directly control the expression of MCT1 and MCT4 (Rastogi et al., 2023). MCT1 and MCT4 can also impact immune cell function and facilitate immunological escape from tumors by altering the pH of the tumor microenvironment (Khatami et al., 2019). According to specific research, lower MCT1 expression in thyroid cancer is linked to elevated methylation of SLC16A1 gene promoters (Silva et al., 2023).

### 2.4 MCTs’ function in lactate transport and acidification of the microenvironment

In thyroid cancer, monocarboxylic acid transporters (MCTs) are crucial for lactate metabolism and microenvironment acidification. Their effects are comparable to those of other tumor types, with a few exceptions. Lactate produced by surrounding stromal cells or immune cells can be absorbed by cancer cells through MCT1 in the thyroid cancer microenvironment. The thyroid cancer cells can use lactate uptake as an energy source to replenish ATP produced by glycolysis (Mackay et al., 2022). This effect is particularly significant in hypoxic or nutrient-deficient microenvironments. According to some research, MCT1 expression and the capacity of thyroid cancer cells to multiply are positively correlated. This is because MCT1 increases lactate utilization, which stimulates cancer cell proliferation (Khatami et al., 2019; Kawamura et al., 2016). Additionally, it has been demonstrated that MCT4 contributes to the movement of lactate generated by thyroid cancer cells to the cell’s outside, preventing intracellular acidification and cell damage (Sandulache et al., 2012; Sulaieva et al., 2020). While the acidic microenvironment can encourage the formation of blood vessels, which increases the supply of nutrients and oxygen for tumor growth (Kim J. et al., 2025), it also suppresses the activity of immune cells, including T cells and NK cells, which helps the immune system escape from tumors (Reinsalu et al., 2025). In thyroid cancer, MCT1 and MCT4 may cooperate to create a “lactate shuttle” (Offermans et al., 2025; Lee et al., 2025), in which the cancer cells absorb lactate produced by the surrounding cells for energy, much like in other tumor forms. Cancer cells create lactate, which is released into the environment and causes acidification. Different subtypes of thyroid cancer may exhibit varying degrees of MCT1 and MCT4 expression; generally speaking, MCT4 expression increases with the degree of malignancy (Jiang T. et al., 2025). It is necessary to conduct additional research to elucidate the rather complex variations in MCT1 expression, which may be connected to the aggressiveness and prognosis of cancers. MCTs are anticipated to be possible targets for therapy since they are crucial for the development and microenvironment control of thyroid cancer. By preventing the expression or activity of MCT1 and MCT4, one can disrupt lactate metabolism and improve the tumor microenvironment to stop the growth and spread of the tumor (Vera et al., 2024; Tang J. et al., 2024).

## 3 Regulatory mechanisms of lactate and lactylation in thyroid cancer

### 3.1 HIF-1α pathway

**FIGURE 1:**
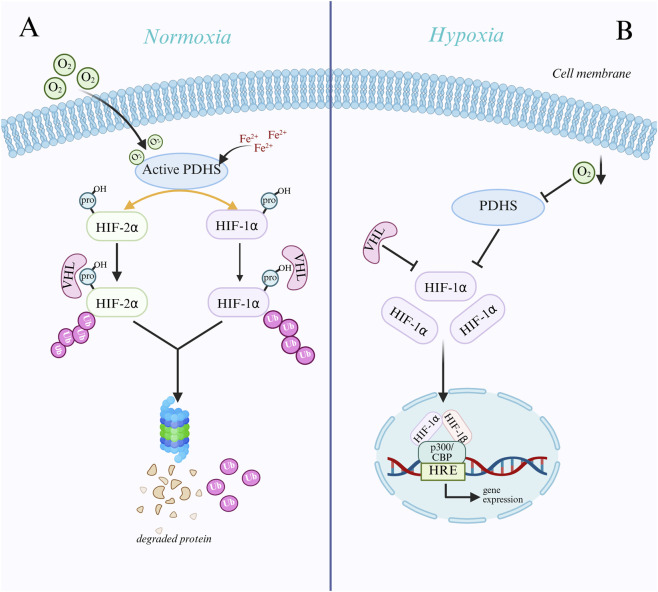
Mechanisms of HIF-α subunit regulation under normoxic and hypoxic conditions. **(A)** Under normoxic conditions, prolyl hydroxylases (PHDs) hydroxylate specific proline residues of the HIF-α subunit using oxygen, Fe^2+^, and α-ketoglutarate (α-KG). Hydroxylated HIF-α is recognized and ubiquitinated by VHL proteins and subsequently degraded via the proteasomal pathway. **(B)** Under hypoxic conditions, where oxygen deprivation results in suppressed activity of PHDs, the HIF-α subunit is not hydroxylated, thus evading recognition and degradation by VHL and stabilizing and accumulating within the cell. Subsequently, HIF-α translocates to the nucleus, forms a heterodimer with HIF-1β, binds to coactivators such as p300/CBP, recognizes and binds to hypoxia response elements (HREs), activates the transcription of downstream genes, and facilitates cellular adaptation to a hypoxic environment.

Using oxygen as a substrate, prolyl hydroxylases (PHDs) catalyze the hydroxylation of specific proline residues on the HIF-α subunit when enough oxygen is present. VHL proteins recognize the hydroxylated HIF-α subunit, which is then ubiquitinated and ultimately broken down by the proteasome pathway (Joharapurkar et al., 2024; Kleibert et al., 2025). Conversely, hypoxia suppresses PHD activity and stops the HIF-α subunit from being hydroxylated, stopping VHL from identifying it. This improves the protein’s stability and causes it to accumulate in cells, ultimately triggering the transcription of target genes (Seymour et al., 2025). With oxygen, α-ketoglutarate, and divalent iron ions as substrates, PHDs are members of the α-ketoglutarate-dependent dioxygenase family. Pro402 and Pro564 are the primary hydroxylation sites of the catalytic substrates HIF-1α, whereas Pro405 and pro531 are the primary hydroxylation sites of catalytic HIF-2α (Joharapurkar et al., 2024). The HIF-α subunit attaches to PHDs after PHDs first bind to Fe2+ and α-KG. Then, oxygen binds to the PHDs’ active center, and PHDs catalyze the proline residues of the HIF-α subunit to undergo hydroxylation, producing hydroxylated HIF-α subunits, succinic acid, and carbon dioxide (Jucht and Scholz, 2024; Lu et al., 2025). Lack of oxygen, ferrous ion deficit, and α-KG concentration will decrease PHD activity and stop them from hydroxylating HIF-α.The α-domain of the VHL protein, an E3 ubiquitin ligase involved in the ubiquitin-proteasome pathway,can identify hydroxylated HIF-α subunits. This enables VHL to attach directly to the ODD domain of hydroxylated HIF-α subunits, starting the proteasome’s ubiquitination and degradation of the subunits (Pauzaite and Nathan, 2024). The proline hydroxylation site of PHD and the region of VHL protein recognition are both impacted by the HIF-α subunit’s oxygen-dependent degradation domain (ODDD) (Saber et al., 2024). Hypoxia inhibits PHD activity and prevents HIF-α from being hydroxylated, which prevents VHL from recognizing and ubiquitinating it. This results in the protein becoming stable and building up intracellularly, triggering the transcription of target genes (Negri, 2022; Figure 1). Research has demonstrated that the target genes HIF-1α regulates in thyroid cancer are crucial for tumor cell metabolism, angiogenesis, cell division, metastasis, and immune evasion (Zhong et al., 2022). For instance, HIF-1α can increase GLUT1 and GLUT3 expression, increase the uptake of glucose by thyroid cancer cells, and provide substrates for glycolysis to meet their energy needs through SLC2A1 (encoding GLUT1) and SLC2A3 (encoding GLUT3) (Yang et al., 2024). The SLC16A3 gene encodes MCT4, and its overexpression can increase lactate excretion, acidify the tumor microenvironment, and create an environment conducive to tumor cell metastasis (Fu et al., 2021; Jiang et al., 2021). The immune checkpoint protein PD-L1, which is encoded by the CD274 gene, can attach to the PD-1 receptor on the surface of T cells. HIF-1α can inhibit T cell function and boost PD-L1 expression, which is detrimental to anti-tumor immunity and encourages tumor immune escape (Nicolini and Ferrari, 2024). A thorough grasp of the HIF-1α signaling pathway’s mechanism of action and its target genes may aid in developing novel therapeutic approaches that block tumor growth and metastasis and target HIF-1α or its downstream target genes, potentially opening up new treatment options for thyroid cancer.

### 3.2 PI3K/AKT/mTOR pathway

**FIGURE 2:**
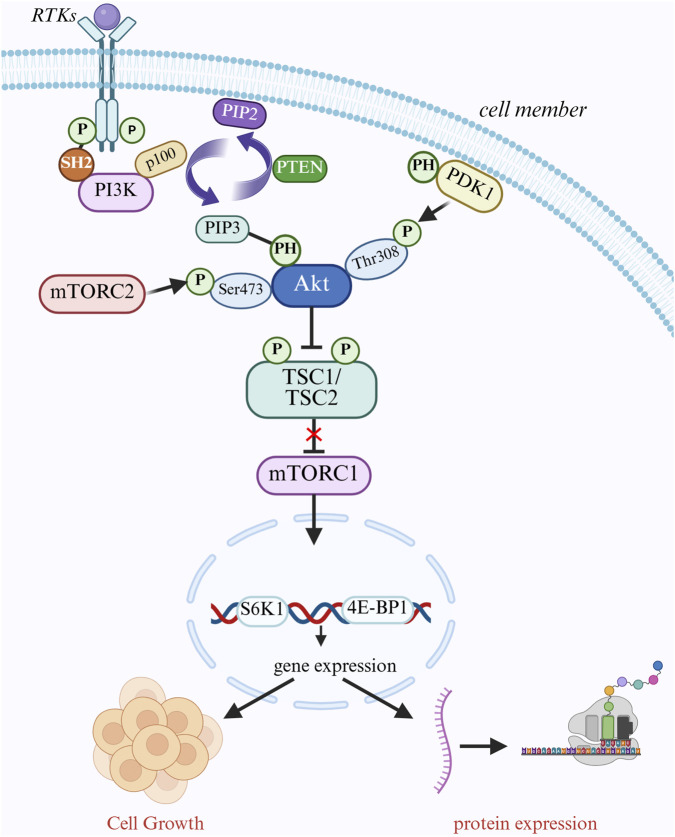
PI3K/AKT/mTOR signaling pathway mediates cell growth regulation. Cell surface receptor tyrosine kinases (RTKs) bind to ligands, dimerize, and activate their kinase domains, which in turn phosphorylate themselves. pI3K is recruited to the cell membrane and binds to phosphorylated RTKs via its SH2 structural domain, which converts PIP2 to PIP3. PIP3 recruits AKT and PDK1 to the cell membrane via its PH structural domain, and PDK1 phosphorylates AKT at Thr308 while mTORC2 phosphorylates AKT at Ser473. PIP3 recruits AKT and PDK1 to the cell membrane through its PH domain. PDK1 phosphorylates the Thr308 site of AKT, while mTORC2 phosphorylates the Ser473 site of AKT, fully activating AKT. activated AKT phosphorylates the TSC1/TSC2 complex, which deregulates mTORC1 and releases mTORC1. mTORC1 promotes protein synthesis and cell growth by phosphorylating S6K1 and 4E-BP1. pTEN converts PIP2 to PIP3 by translating PIP3 back to PIP1. PTEN negatively regulates this pathway by converting PIP3 back to PIP2.

The proper ligands (growth factors) are attached to cell surface growth factor receptors (RTKs) at the onset of signaling, including platelet-derived growth factor receptor (PDGFR) and epidermal growth factor receptor (EGFR) (Haxho et al., 2016). When RTKs bind to ligands, they dimerize and activate intracellular kinase domains, which leads to receptor autophosphorylation (Staniek and Rizzi, 2025). The phosphotyrosine residue on phosphorylated RTKs binds to the SH2 domain of PI3K, attracting PI3K close to the cell membrane and being activated by the RTKs, catalyzing the conversion of PIP2 to PIP3 on the cell membrane (Rahman et al., 2025; Figure 2). In thyroid cancer, particularly follicular carcinoma, the catalytic subunit p110α encoding PI3K, encoded by the PIK3CA gene, is frequently mutated, which results in constitutive activation of PI3K and changes the conformation of its domain, increasing its catalytic activity (Qiang et al., 2025). It may stay activated even when no ligands are present, which leads to ongoing activation of downstream signaling pathways. Second, the PH domain of the AKT protein can be identified and attached to PIP3 to bring AKT to the cell membrane. PIP3 can also bring PDK1, which has a PH domain, to the cell membrane, which phosphorylates the 308-position of AKT (Thr308) (Maharati et al., 2024). AKT relieves the inhibition of mTORC1 by phosphorylating and inhibiting the TSC1/TSC2 complex, and to encourage protein synthesis, active mTORC1 phosphorylates downstream substrates S6K1 and 4E-BP1 (Fontana et al., 2024). Additionally, the Mtorc2 complex phosphorylates serine at site 473 of AKT (Ser473), which is fully activated after double phosphorylation and becomes an important cell signaling center (Sicurella et al., 2025). By phosphorylating apoptosis-related proteins like BAD and BAX, AKT can prevent apoptosis and promote tumor cell growth (Parry et al., 2021). Abnormal activation of this system has been connected to several cancers, such as colorectal, stomach, and breast cancers, and it has been shown that blocking this pathway results in tumor regression.

### 3.3 MAPK/ERK pathway

The MAPK family includes the serine/threonine protein kinase ERK, of which ERK1 and ERK2 are the most extensively researched members. ERK is primarily activated by the cascade reaction of Ras protein and MAPKKK (Yuan et al., 2024). Phosphorylated ERK is transported from the cytoplasm to the nucleus, which controls the activity of different transcription factors, thereby influencing gene expression (Toye et al., 2024). The Jun and Fos proteins make up AP-1, and ERK1/2 can phosphorylate and activate the Ser63 and Ser73 sites of the Jun protein and the Thr325 site of the Fos protein (Cho et al., 2009). These phosphorylation events improve AP-1’s ability to bind to target genes, including the crucial enzyme HK2 (hexokinase 2) in glycolysis. ERK1/2 can directly phosphorylate HK2 to increase its activity and stability and improve HK2’s binding to the outer membrane of the mitochondria. This makes it easier for HK2 to obtain APT produced by mitochondria, increases its catalytic efficiency, and supplies more energy sources for tumor growth (Jiang et al., 2022). According to specific research, AP-1 and HIF-1α frequently cooperate to activate LDHA transcription and increase tumor cells’ ability to produce lactate (Wang Q. et al., 2025). Under normoxic conditions, the ERK1/2 pathway can also phosphorylate the Ser641 and Ser643 sites of HIF-1α, increase its transcriptional activity, and promote its binding to cofactors like p300/CBP(He et al., 2025). It can also control its expression by influencing the translation and stability of HIF-1α, which controls important rate-limiting enzymes in glycolysis, including PFK1 (phosphofructokinase 1) (Yu et al., 2021). Of the three primary isoforms of PFK1, ERK1/2 can phosphorylate the Ser529 site of PFK1-M, changing its conformation to facilitate substrate binding and leading to the buildup of glycolytic intermediates (Fernandes et al., 2020). This is a common occurrence in tumor cells. According to specific research, MAPK/ERK and other signaling pathways might have a cross-effect. For example, MAPK/ERK and PI3K/AKT frequently cooperate to activate mTOR and encourage the translation of glycolytic enzymes (Gulec et al., 2024). They also increase the expression of glycolysis-related genes by co-activating transcription factors like HIF-1α and c-Myc (Wang S. et al., 2024; Wang H. et al., 2024). AMPK is a cellular energy sensor activated in energy deprivation and slows energy-consuming metabolic processes (Wang et al., 2021; Icard et al., 2021). It also decreases the translation of glycolytic enzymes. Nevertheless, MAPK/ERK and AMPK have opposing actions.

### 3.4 Hypoxia’s impact on lactate production

HIF is the primary regulator of lactate generation by oxygen, and its most extensively researched subunit is HIF-1α (Masoud and Li, 2015). The hydroxylation of proline residues in the HIF-α subunit is catalyzed by proline hydroxylases (PHDs) when sufficient oxygen is available. The hydroxylated HIF-α subunit is identified and ubiquitinated by VHL proteins, which are then broken down by the proteasome pathway (Mescht et al., 2025). Inhibitors (FIHs) catalyze the hydroxylation of the HIF-α subunit’s asparagine residues, which stops the transcriptional cofactor p300/CBP binding (Lee et al., 2024). When hypoxia occurs, PDH and FIH activity are suppressed, the HIF-α subunit stabilizes, and the HIF-α subunit enters the nucleus by forming a heterodimer complex with the HIF-β subunit (Strowitzki et al., 2019),attaching to the hypoxic response element (HRE) on the LDHA gene’s promoter region (Rankin and Giaccia, 2008). The transcriptional cofactor p300/CBP is recruited by the HIF-1α/HIF-1β complex bound to the LDHA promoter region. A loose chromatin structure can be caused by p300/CBP’s histone acetyltransferase activity, which can acetylate neighboring proteins. Acetylated chromatin facilitates RNA polymerase II binding and transcriptional initiation, promoting the transcription of LDHA genes and raising lactate production (Wu et al., 2025). Hydrogen ions will be released as lactate builds up, lowering the pH both within and outside the cell and preventing enzyme activity inside the cell, affect the normal function of the cell and even lead to cell death (Chen J. et al., 2025),however, monocarboxylic acids (such lactate and pyruvate) must be transported from intracellular to extracellular spaces or the other way around via MCTs (monocarboxylic acid transporters), MCTs transport lactate in a way that co-transports hydrogen ions. This means that MCTs transport lactate molecules and hydrogen ions, thereby promoting the reduction of intracellular acidity and extracellular acidification, protecting cells from damage to the acidic environment and leading to acidification of the tumor microenvironment. In order to help cells adapt to the hypoxic environment, HIF-1α activates LDHA to boost lactate synthesis and MCT4 to improve lactate transport. This helps cells grow, metastasize, and escape from the immune system (Pan et al., 2024; Rong et al., 2024).

### 3.5 Impact of an acidic environment on the functioning of metabolic enzymes

Phosphofructokinase 1 (PFK1) catalyzes an irreversible step in the glycolytic pathway, and its activity is carefully regulated by a number of factors, including substrates, products, energy states, and pH levels. Because PFK1 is extremely sensitive to pH, its active center contains some important histidine residues. These histidine residues are typically protonated in an acidic environment, changing the enzyme’s conformation, decreasing its ability to bind to the substrate fructose-6 phosphate, and inhibiting its catalytic activity (Bartrons et al., 2018). Studies have demonstrated that tumor cells can selectively express acid-insensitive PFK1 isoforms, which can maintain high activity even in acidic environments, guaranteeing glycolytic flux. Tumor cells have evolved several adaptation and compensatory mechanisms to maintain glycolytic flux in the acidic tumor microenvironment (Bartrons et al., 2018; Kotowski et al., 2021). Proton pumps and sodium hydrogen exchangers (NHEs) are also expressed by tumor cells to expel hydrogen ions from the cell, raising the pH level. This can lessen the acidic environment’s inhibitory effect on PFK1 and allow it to operate normally (Bernardazzi et al., 2022; Pukkanasut et al., 2024). Because PFK1 is a rate-limiting enzyme, tumor cells compensate for the decreased activity of PFK1 by upregulating the expression of the downstream crucial enzyme PKM2, which increases the efficiency of glycolysis (Pinweha et al., 2016). When PFK1 activity is suppressed, glucose-6-phosphate (G-6-P), an upstream metabolite of PFK1, is capable of being transformed into ribose-5-phosphate and NADPH via the pentose phosphate pathway (PPP), which gives tumor cells the raw materials they need for biosynthesis (Campos and Albrecht, 2023). PKM1 and PKM2 are the two primary isomers of pyruvate kinase (PK), which catalyzes the final step of glycolysis, the conversion of phosphoenolpyruvate (PEP) to pyruvate. PKM2 has a high catalytic activity and tends to form tetramers; the tetrameric form is typically unstable in tumor cells and readily breaks down into dimers with low activity (Yang et al., 2025). F-1,6-BP(Fructose-1,6-bisphosphate) is a crucial PKM2 activator. Through the upregulation of upstream glycolytic enzymes (such as HK and PFK1), tumor cells speed up the conversion of glucose to F-1,6-BP(Fructose-1,6-bisphosphate). The pyruvate generated by glycolysis is more likely to be converted to lactate than to reach the mitochondria for aerobic oxidation, even if PKM2 activity declines. Lactate synthesis can also recycle NAD+ and sustain glycolysis (Liao et al., 2024; Gao et al., 2022). To adapt and compensate for decreased PKM2 activity brought on by acidic surroundings, tumor cells also decrease intracellular acidity by controlling downstream metabolic pathways to divert G6P to the pentose phosphate pathway and increasing proton pump activity (Ahamed et al., 2023). Through the “open source” and “throttling” techniques, tumor cells can increase glycolytic flux and ATP production while reducing energy consumption and responding to changes in the metabolic environment by altering metabolic pathways, controlling PH levels, and encouraging lactate creation.

### 3.6 Lactylation modifies regulatory enzymes

**FIGURE 3:**
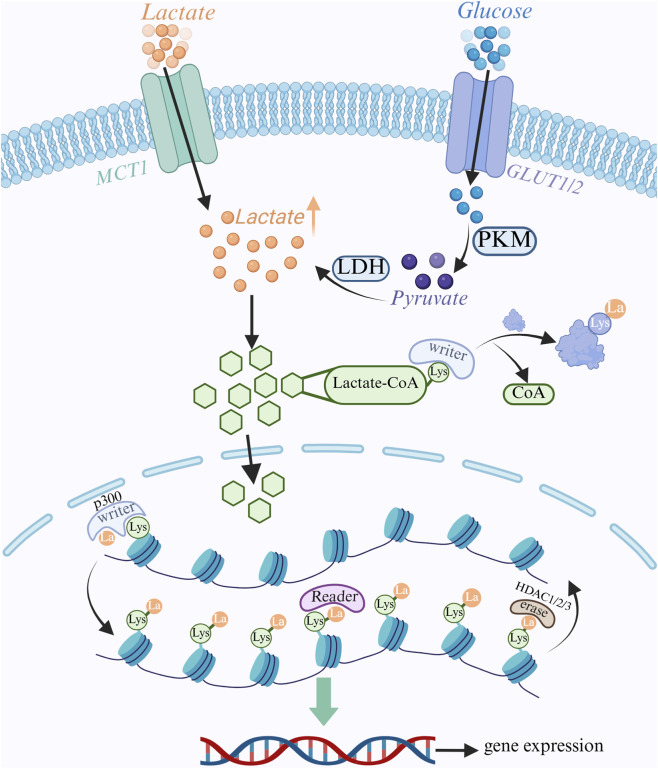
Lactate metabolism and epigenetic regulation. Glucose enters the cell via the GLUT transporter protein and undergoes glycolysis to produce pyruvate. Pyruvate is converted to lactate by LDH. Lactate enters the cell via MCT1 transporter. Intracellular lactate is converted to lactyl coenzyme A, which serves as a substrate for lactylation modification. Lactate modification is catalyzed by “Writer” enzymes (e.g., p300), which transfer lactate groups to lysine residues on histones and non-histone proteins to regulate gene expression; “Eraser” enzymes (e.g., HDAC1/2/3), which remove lactate groups; and “Reader” enzymes (e.g., HDAC1/2/3), which remove lactate groups from lysine residues on histones and non-histone proteins. groups; “Reader” proteins recognize lactate modifications and further regulate downstream signaling.

Although lactate is a byproduct of energy metabolism, it has recently been discovered to be a significant signaling molecule and epigenetic modifier that regulates gene expression through a process known as lactylation modification, in which a lactate group is covalently attached to a protein’s lysine residue (Zhang W. et al., 2025). Lactylation modifications are dynamically controlled by a group of enzymes that are principally in charge of “writing” (introducing lactylation), “erasing” (removing lactylation), and “reading” (recognizing lactylation), much like common post-translational modifications (PTMs), which control a variety of biological processes within cells by changing the structure and function of proteins (Lv et al., 2025). Preliminary study indicates that in situations high in lactate, especially those involving exercise, inflammation, and the tumor microenvironment, lactate can be converted into lactyl-CoA (lactic CoA). Additionally, p300 can bind lactic CoA to lysine residues, which subsequently catalyzes the transfer of lactate groups in lactate CoA to the ε-amino group of lysine, producing lactated modified proteins and coenzyme A as a byproduct (Zeng et al., 2023). P300 activity is regulated by a number of factors, such as enhanced glycolysis, higher lactate concentration, and activation of particular inflammatory signaling pathways (Wang S. et al., 2025). HDACs are a group of enzymes that eliminate histone acetyl groups, and HDAC1, HDAC2, and HDAC3 have been found to remove lactate groups from histones, thereby reversing lactylation modifications (Zhang Y. et al., 2025; Figure 3), they typically use zinc ions as catalytic centers to hydrolyze amide bonds on lysine residues to release lactate molecules (Biersack et al., 2025). According to some research, certain HDACs can restrict the expression of genes linked to inflammation and lessen inflammatory reactions.

In contrast, other HDACs have tumor-suppressive properties or encourage the growth of tumors. However, the precise chemical process that underlies HDACs’ substrate selectivity for lactylation modification might differ from acetylation (Leotta et al., 2025; Lin et al., 2025; Liu Y. et al., 2025). Studies on “readzyme” for lactylation modifications are comparatively rare, and although some research indicates that proteins may bind to lactated lysine preferentially, domains or proteins that specifically recognize and bind lactylation changes have not been identified. The writing enzyme (p300) and erase enzyme (HDAC1/2/3) work together to govern lactylation modification. This dynamic regulatory mechanism is crucial for many biological processes, including gene expression, cell metabolism, inflammatory response, and carcinogenesis. The currently known enzymes that govern lactylation are scarce; therefore, identifying additional regulators is essential to strengthen the lactylation-modification network.

### 3.7 Lactylation modification and gene expression regulation

The lysine residues of histones (like H3K18la and H3K9la) are the primary site of lactylation modification (Wang F. et al., 2025). These changes lessen the interaction between histones and negatively charged DNA and neutralize the charge of histones (W. Zhao et al., 2025), creating a more accessible open chromatin state (Yang Y. et al., 2024). This makes it easier for transcription factors and other regulatory proteins to bind, which in turn makes gene transgenesis easier to initiate. However, it has been discovered that certain transcription factors possess lactate-binding domains that can be attracted to particular gene loci and change the transcription factors’ conformation (Wang F. et al., 2025; Zhang X. et al., 2024),which impacts their ability to bind DNA or interact with other proteins. High levels of glycolysis and lactate are frequently observed in the tumor microenvironment (Li J. et al., 2024), which supplies enough substrates for the formation of lactylation modifications. These modifications are used by tumor cells to control gene expression and encourage tumor growth, metastasis, and drug resistance (Zhang Y. et al., 2024; Lv et al., 2025). The high lactate environment in certain tumor cases, like colorectal cancer (CRC) cells, causes an increase in H3K18 lactylation. H3K18la modification enriches on the promoters of genes linked to tumor metastasis, promoting the expression of these genes and improving the ability of CRC cells to spread (Chen B. et al., 2024). Lactate causes H3K9 lactylation alteration in hepatocellular carcinoma (HCC) cells, which activates the Wnt/β-catenin signaling pathway and causes tumor cells to proliferate and spread (Lu et al., 2024). Furthermore, it has been discovered that in certain instances of breast cancer, triple-negative breast cancer (TNBC) cells exhibit increased glycolytic activity, which leads to the buildup of lactate within the cells. Consequently, this promotes histone H3K18 lactylation, which leads to FOXM1 expression (Lv et al., 2025; Gui et al., 2024), thereby augmenting the growth, migration, and invasion of cancer cells. Furthermore, we have discovered that lung cancer cells decrease T cell activity, facilitate immunological escape of tumors, and regulate immune checkpoint marker expression (like PD-L1) by lactylation modification (Ding et al., 2025; Wang W. et al., 2025). Even in aerobic environments, glycolysis is preferentially carried out in thyroid cancer, particularly papillary thyroid cancer (PTC) (Fukushi et al., 2022; Jiang H. et al., 2025), generating copious amounts of lactate that can be utilized as an energy source as well as take part in lactylation modifications that impact their gene expression. Although studies on the effects of lactylation on thyroid cancer are still in their infancy, some suggest that lactate may boost the expression of specific genes associated with the epithelial-mesenchymal transition (EMT) in thyroid cancer cells (Xu et al., 2024), which would increase the tumor cells’ capacity to spread. It is worthwhile to look into whether lactylation alteration affects how sensitive thyroid cancer cells are to radioactive iodine. Research has even demonstrated that the metabolic state of tumor cells may influence the uptake and therapeutic effect of radioactive iodine (Shen et al., 2025; Chen P. et al., 2024). Thus, we hypothesize that lactylation alteration might be a significant factor in this, and further investigation into it should yield new targets and approaches for tumor diagnostics and treatment.

## 4 Lactate’s function in the microenvironment of thyroid cancer

### 4.1 Lactate’s function in tumor angiogenesis

**FIGURE 4:**
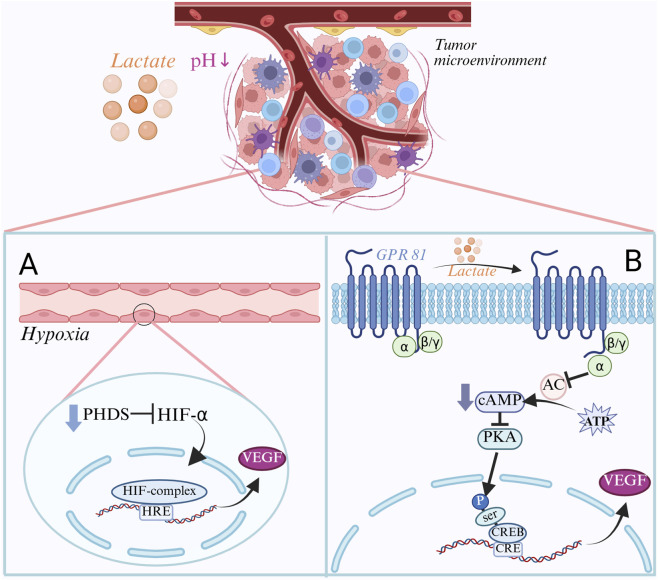
Regulatory mechanisms of lactate in tumor angiogenesis. **(A)** Hypoxia-induced angiogenic pathway: In the tumor microenvironment, lactate accumulation leads to a decrease in pH, which in turn leads to an insufficient supply of oxygen. Under hypoxia, PHDs activity is inhibited, HIF-1α is stabilized and forms a complex with HIF-1β. This complex binds to the HRE in the promoter region of the VEGF gene and promotes VEGF transcription, thereby inducing angiogenesis. **(B)** GPR81-mediated signaling pathway: Lactate acts as an endogenous ligand that binds to GPR81 and activates downstream signaling. GPR81 activation inhibits adenylyl cyclase (AC) activity, which decreases cAMP levels and thus inhibits PKA activity. This promotes phosphorylation of CREB, which binds to the CRE in the promoter region of the VEGF gene and recruits transcriptional cofactors to promote VEGF transcription, thereby inducing angiogenesis.

Angiogenesis is a vital step in development and metastasis, delivering oxygen and nutrition to tumor cells and allowing them to enter the blood circulation, producing distant metastases. As a result of glycolysis of tumor cells, lactate plays a crucial role in tumor angiogenesis (Yao et al., 2025). Glycolysis produces pyruvate, which tumor cells often convert to lactate even when there is enough oxygen present. Lactate is then transported outside the cell by monocarboxylic acid transporters (MCTs), which causes the tumor microenvironment to become more acidic (Duan et al., 2022). The acidic environment lowers the affinity of hemoglobin for oxygen, resulting in a decrease in the ability of oxygen to be released into tumor tissues, resulting in vascular dysfunction, diminished oxygen delivery, and harm to the tumor blood vessels’ endothelial cells (Doherty and Cleveland, 2013). Under hypoxic conditions, PHD activity is inhibited and the HIF-1α protein is stabilized. After then, the stable HIF-1α joins HIF-1β to create the HIF-1α/HIF-1β complex. It identifies and attaches itself to the angiogenesis factor gene’s promoter region’s hypoxia response element (HRE), promoting the transcription of the VEGF gene (Polet and Feron, 2025; Yang et al., 2019). Tumour-derived VEGF binds to VEGF receptors on endothelial cells, triggering downstream signalling that fosters tumour-cell proliferation. This process secures oxygen and nutrient supply through endothelial migration and lumen formation, while simultaneously promoting lactate production—together establishing a self-reinforcing malignant loop that perpetuates tumour progression (Yang et al., 2019; Chen et al., 2022). According to research, hypoxia-induced HIF-1α activation can boost the expression of VEGF as well as other angiogenesis factors like FGF and PDGF. The combined action of these angiogenesis factors eventually results in the development of tumor blood vessels (Xu and Tang, 2024). Furthermore, instead of activating HIF-1α, lactate might either directly or indirectly stimulate angiogenesis through alternative routes. Cellular function can be controlled by lactate’s ability to bind to GPR81 (G protein-coupled receptor 81) and trigger downstream signaling cascades (Liu H. et al., 2024). GPR81 is a natural binding target and an endogenous ligand for lactate (Zhang T. et al., 2024). GPR81 expression is crucial in vascular endothelial cells’ reaction to lactate. GPR81 is primarily linked to the Gi/o protein, which is activated when lactate binds to it. The Gi/o protein then splits into α subunits and βγ dimers (Ristic et al., 2017), whereby the Giα subunit inhibits protein kinase A (PKA) action, decreases intracellular cAMP levels, and suppresses adenylyl cyclase (AC) function, and promotes phosphorylation of the cAMP response element-binding protein’s (CREB) Ser133 site (Payen et al., 2019). The cAMP response element (CRE) in the VEGF gene initiation region is recognized and bound by the phosphorylated CREB protein, and the p-CREB protein that binds to CRE recruits transcriptional cofactors (such as CBP and p300) that have histone acetyltransferase (HAT) activity and can acetylate nearby histones, resulting in a loose chromatin structure that favors RNA polymerase II binding and transcriptional initiation, thereby promoting transcription of the VEGF gene, eventually, the angiogenic factor VEGF was substantially expressed (Ishihara et al., 2022; Sun et al., 2017; Ristic et al., 2017; Figure 4). According to certain research, GPR81 activation might indirectly trigger the MAPK/ERK signaling pathway, which can regulate the reorganization of the cytoskeleton and thus promote cell migration (Shang et al., 2023). GPR81 can interact with endothelial and tumor cells to create a positive feedback loop that encourages angiogenesis and tumor growth.

### 4.2 Lactate’s inhibitory influence on immune cell function

T cell activation is essential for immune response adaption as well as for controlling infections, treating cancer, and creating immunological memory. The antigen is captured, processed, and bound to major histocompatibility complex (MHC) molecules in the form of peptides by antigen-presenting cells (APCs), T cell receptors specifically attach to MHC-peptide complexes on the surface of APCs, and costimulatory molecules on APCs attach to costimulatory receptors on T cells, initiating T cell signaling pathways that activate T cells (Shi et al., 2025). In order to support cell proliferation, activated T cells need a quick energy source. Compared to resting T cells, activated T cells greatly increase glycolysis to meet their energy needs (Kim et al., 2025). The experiment demonstrated that lactate could directly inhibit the glycolysis of T cells, which lowers the production of ATP and inhibits the proliferation of T cells (Kim J. et al., 2025; Ma et al., 2025; Liu Q. et al., 2025). To measure the T cells’ ATP level and glycolysis rate, the researchers cultivated peripheral blood T cells in various lactate concentrations (0 mM, 10 mM, and 20 mM). They discovered that as lactate concentration rose, T cells’ ATP levels and glycolysis rates dramatically dropped, decreasing by 50% and 40% in the group supplemented with 20 mM lactate, respectively, in comparison to the control group without lactate. Lactate can enter mitochondria as a substrate for oxidative phosphorylation, which is another crucial process for T cell energy metabolism in addition to glycolysis (Jiang, 2020). I Researchers cultivated mouse T cells in various lactate concentrations and then looked at the mitochondrial respiration rate and functional markers of the T cells in animal experiments. They found that as lactate concentrations increased, the mitochondrial respiration rate of the T cells increased, but that T cell expression depletion markers like PD-1 and Tim-3 also increased (Jiang, 2020; Wu and Zhang, 2025). Experiments have shown that lactate can promote oxidative phosphorylation of T cells, but it may also induce T cell exhaustion, reduce their proliferative ability and anti-tumor activity, because excessive oxidative phosphorylation can lead to mitochondrial dysfunction, produce excessive ROS, and damage T cells Jiang M. et al., 2024). Some studies have also demonstrated that lactate can block the expression of perforin and granzyme in CTLs, impair the cytotoxic function of CTLs, reduce their ability to destroy tumor cells, and so enhance the immunological escape of tumors (Hao et al., 2024; Liu Q. et al., 2025). By releasing inhibitory cytokines and interacting with effector T cells via molecules like CTLA-4. In the thyroid cancer microenvironment, treg cells reduce the immune system’s ability to destroy cancer cells by inhibiting the function of cytotoxic T lymphocytes (CTLs) (Huo et al., 2024). Research has demonstrated a correlation between the infiltration density of Treg cells in thyroid cancer tissues and poor prognosis, tumor stage, and lymph node metastasis (R. Shen et al., 2020; Pasquali et al., 2023). IL-10 is an inhibitory cytokine secreted by Treg cells, which can inhibit the activation of antigen-presenting cells (APCs), suppress the function of cytotoxic T lymphocytes (CTLs) and lower the production of cytokines (El-Shemi et al., 2025). Studies have shown that lactate can influence cytokine expression by changing intracellular signaling pathways and transcription factor activity, which weakens the immune response (Jin et al., 2018). Treg cells have significant expression of the immunological checkpoint molecule CTLA-4 (cytotoxic T lymphocyte-associated protein 4). T cell activation is suppressed, APC is prevented from costimulatory signaling T cells, CD80/CD86 molecules from antigen-presenting cells are bound, and CD28’s binding to CD80/CD86 is competitively inhibited (Inoue et al., 2025; Carbone et al., 2024). Lactate acidifies the tumor cell microenvironment, which inhibits the anti-tumor immune response, influences CTLA-4 production via the EPAC1 signaling pathway, and strengthens its capacity to prevent effector T cell activation (Fischbeck et al., 2020).

### 4.3 Interaction of lactate with fibroblasts

**FIGURE 5:**
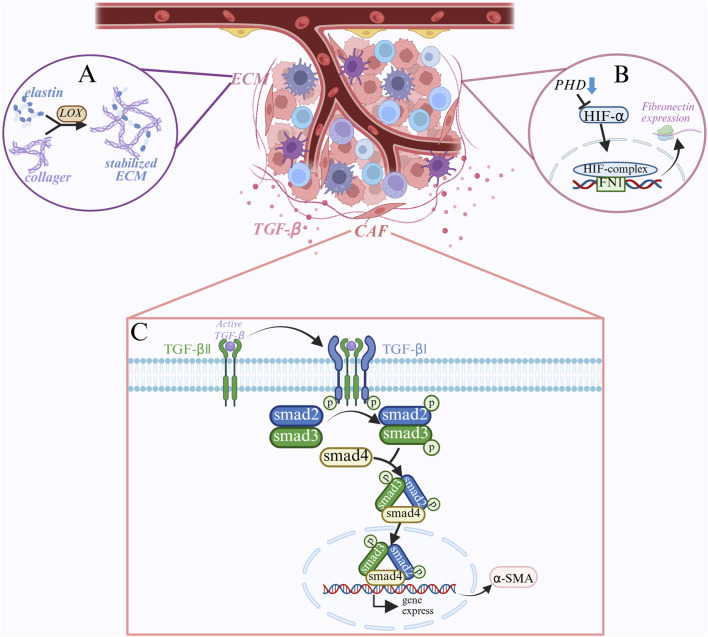
Mechanisms of lactate in the tumor microenvironment: **(A)** lactate enhances ECM stability by promoting LOX expression, which catalyzes collagen cross-linking to form a stable ECM that provides structural support for tumor cells. **(B)** Under hypoxic conditions, PHDs are less active, HIF-1α stabilizes and forms active HIF-1 with HIF-1β, which promotes the expression of fibronectin and increases ECM composition. **(C)** TGF-β signaling pathway activates fibroblasts. TGF-β binds to TGFBRII, activates TGFBRI, phosphorylates Smad2/3, forms Smad complex, induces the expression of α-SMA, and induces the differentiation of fibroblasts to CAFs, which enhances tumor invasion and metastasis.

Normal fibroblasts are mainly involved in tissue repair and the maintenance of extracellular matrix (ECM) homeostasis. Additionally, transforming growth factor β (TGF-β), one of the most important activators of cancer-associated fibroblasts (CAFs), can be released in high quantities by tumor cells in the tumor microenvironment (Chen X. et al., 2025; Leonardo-Sousa et al., 2025). TGF-β normally exists in a latent state and needs to be activated to bind to TGF-β receptors on the cell surface (Jing H. et al., 2025). TGF-β receptor II (TGFBRII) is phosphorylated when TGF-β binds to it, and this attracts TGF-β receptor I (ALK5) (Ungefroren et al., 2018). The intracellular signaling molecules Smad2 and Smad3 are further phosphorylated by ALK5 following its activation by TGFBR II phosphorylation. The phosphorylated Smad2/3 then combines with Smad4 to deliver it to the nucleus (Jeganathan et al., 2017). To regulate the expression of specific genes, such as α-SMA (α-smooth muscle actin), the Smad complex interacts to other transcription factors, coactivators, or corepressors inside the nucleus (Hong et al., 2025). TGF-β stimulates the production of α-SMA, increases CAF contractility, and encourages tumor cell invasion and metastasis. α-SMA is a signature protein of CAF that plays a role in matrix remodeling and cell contraction (Jia et al., 2025). In order to detect TGF-β expression, Smad phosphorylation level, and α-SMA expression, fibroblasts were cultured under varying concentrations of lactate in fibrosarcoma cells. The results demonstrated that lactate could directly induce α-SMA expression and promote the differentiation of fibroblasts into CAFs by activating the TGF-β signaling pathway, indicating that lactate can increase TGF-β expression in a dose-dependent manner, Smad phosphorylation, and α-SAM(Chen W. et al., 2024; Khiat et al., 2025; Figure 5). Additionally, by employing the α-SAM antibody for immunohistochemical staining, it was discovered that the number of CAFs in thyroid cancer tissues was much larger than that in normal thyroid tissues, and that the number of CAFs was positively connected with the tumor’s aggressiveness and metastasis (Li et al., 2022; Wang Y. et al., 2024). Furthermore, CAFs can secrete large amounts of extracellular matrix (ECM) components, such as collagen and fibronectin. It has been discovered that activation of HIF-1α can also promote the transcription of collagen and fibronectin, in addition to TGF-β activating the Smad signaling pathway to regulate gene transcription associated with ECM (Yang et al., 2021). High lactate and hypoxia cause PHDs (proline hydroxylase) to become less active, HIF-1α to accumulate and stabilize since it is not hydroxylated, and the stable HIF-1α and HIF-1β to form heterodimers to produce the active transcription factor HIF-1. Following recognition and binding of the target gene’s promoter region’s core sequence 5′-RCGTG-3′, HIF-1 enlists the transcriptional coactivator p300/CBP. This promotes chromatin opening in the promoter region and starts the target gene’s transcription. Increased collagen synthesis is the result of HIF-1’s ability to bind to several collagen gene promoter regions, including COL1A1 and COL1A2 (Xu et al., 2022). Additionally, HIF-1 increases fibronectin expression by binding to the fibronectin gene’s promoter region (FN1) (Assidicky et al., 2022). HIF-1 encourages the deposition of extracellular matrix (ECM), which gives tumor cells structural support, by boosting the synthesis of collagen and fibronectin. Lysine oxidase (LOX), a copper-dependent enzyme, catalyzes the oxidation of lysine residues in collagen and elastin and the creation of aldehyde groups that can cross-link to maintain the extracellular matrix (Nourian et al., 2023). The LOX gene’s promoter region also contains HRE, which HIF-1α can directly bind to promote LOX gene transcription. Higher LOX mRNA results in higher synthesis of LOX protein, and higher LOX activity causes collagen and elastin molecules to cross-link, increasing the ECM’s stability and stiffness (Nourian et al., 2023; Liu M. et al., 2024; Bigos et al., 2024).

## 5 Clinical relevance of thyroid cancer and lactate

### 5.1 Different thyroid cancer subtypes have different lactate levels

The tendency of tumor cells to produce energy through glycolysis is explained by the Warburg effect, which causes lactate to build up even in the presence of plenty of oxygen. Lactate has been implicated in thyroid cancer occurrence, development, metastasis, and treatment resistance in a growing number of investigations in recent years (Feng et al., 2024; Cong et al., 2024; Wang S. et al., 2024). By acidifying the tumor microenvironment, lactate can not only aid in anabolism by giving tumor cells energy and a material foundation for their quick proliferation, but it can also suppress immune cell activity and encourage tumor cell immune escape. Lactate can simultaneously stimulate the development of tumor blood vessels, supply tumor cells with oxygen and nutrition, and cause vascular endothelial growth factor (VEGF) to be expressed. The various histological subtypes of thyroid carcinoma differ substantially in their genetic composition, metabolic mechanisms, and clinical features. Consequently, they might also display distinct traits concerning lactate metabolism. The most prevalent kind of thyroid cancer, papillary thyroid carcinoma (PTC), typically has a favorable prognosis. On the other hand, advanced PTCs and high-level subtypes (such hypercellular and columnar) are more aggressive. LDHA expression in PTC tissues has been found to positively correlate with tumor size, lymph node metastasis, and TNM stage in numerous studies. Additionally, *in vitro* experiments have demonstrated that overexpression of LDHA stimulates PTC cell migration, invasion, and proliferation (Feng et al., 2024; Wang S. et al., 2024). This implies that the development of PTC is significantly influenced by LDHA-mediated lactate generation. Compared to PTC, follicular thyroid carcinoma (FTC), the second most prevalent subtype, has a higher propensity to produce hematogenous metastases. Despite the paucity of research on FTC lactate levels, what is known indicates that FTC cells also have a propensity for increased glycolysis and lactate buildup (Zhao et al., 2020; Addie et al., 2020). Though its regulatory mechanism needs to be further established, mutations in the RAS gene in FTC may be more significant in glycolytic regulation than in PTC, and the expression of specific transcription factors (such HIF-1α) may be regulated, impacting lactate generation (Klaus et al., 2018). Although studies have shown that MTC cells also have a tendency to produce energy through glycolysis, which results in lactate accumulation, unlike PTC and FTV, glycolysis of MTC may be regulated by RET gene mutations, and the tumor microenvironment in MTC, such as hypoxia, further encourages lactate production (Werner et al., 2019; Kanekura, 2023). The parafollicular cells of the thyroid gland are the source of medullary thyroid cancer (MTC), which has the ability to release calcitonin. Undifferentiated thyroid cancer (ATC) cells frequently exhibit significant metabolic reprogramming; two important characteristics of ATC cells are increased glycolysis and lactate buildup (Li Y. et al., 2022), which are directly linked to the cells’ rapid growth, metastasis, and resistance to treatment (Suh et al., 2019). Glycolysis and lactate accumulation are jointly promoted by mutations in genes like TP53 and BRAF, which are frequently found in ATC (Suh et al., 2019; Arciuch et al., 2011). Furthermore, some research has demonstrated that ATC cells have a much higher rate of glycolysis than other thyroid cancer cells, and that stopping glycolysis can dramatically reduce ATC cell growth and metastasis (Li Y. et al., 2022; Sandulache et al., 2012). New concepts and targets for precision therapy can be found by researching the lactate metabolism traits of various thyroid cancer subtypes and creating focused treatment plans.

### 5.2 Inhibitors of LDH

As a crucial enzyme in the glycolytic system, lactate dehydrogenase (LDH) is considered a possible target for tumor therapy. Numerous LDH inhibitors have been developed, and preclinical and clinical studies have shown some anti-tumor effects. Lactate dehydrogenase A (LDHA) is a subtype of the LDH enzyme that is highly expressed in many tumor cells. GNE-140 functions as a small molecule inhibitor that attaches itself to the LDHA enzyme’s active site to prevent the substrate pyruvate from binding to the coenzyme NADH (Ždralević et al., 2018). Depending on how GNE-140 interacts with the enzyme, this inhibition can be competitive, noncompetitive, or mixed (Tufail et al., 2024). GNE-140 lowers lactate synthesis by blocking the enzymatic activity of LDHA, which stops pyruvate from being converted to lactate (Zhao et al., 2024). According to earlier research, GNE-140 demonstrates potent anti-tumor activity against a variety of tumor cell lines, including those from lymphoma, lung, and breast malignancies. It can also cause apoptosis and stop cell division (Mazzio et al., 2021; Li J. et al., 2024). GNE-140 has also been discovered to work in conjunction with chemotherapy medications like doxorubicin and cisplatin in certain clinical treatments, which can improve the chemotherapy impact and go beyond the tumor cells’ resistance to chemotherapy (Varma et al., 2021; Li G. et al., 2024). Targeting lactate metabolism, particularly with LDH inhibitors, may offer a novel approach to treating thyroid cancer, as various subtypes of the disease—particularly ATC—display the traits of glycolysis and lactate accumulation. Additionally, some research has indicated that LDHA inhibitors in conjunction with BRAF inhibitors have demonstrated notable anti-tumor activity in ATC therapy (Gao et al., 2019; Frank et al., 2024). Although GNE-140 has not yet been the subject of clinical trials especially for thyroid cancer, it has demonstrated good safety and efficacy in clinical trials for other solid tumors, and future exploratory research in thyroid cancer patients may be feasible. FX11 is also a lactate dehydrogenase inhibitor; when it binds to the LDH enzyme, it takes up the binding site of NAD+, which stops NAD+ from binding to LDH. This leads to a direct enzymatic inhibition, which stops LDH from catalyzing the conversion of pyruvate to lactate and oxidizing NADH to NAD+(Alobaidi et al., 2023; Wu H. et al., 2021). FX11 suppresses both LDHA and LDHB, in contrast to GNE-140, which may have a wider effect on several tumor cell types. FX11’s inhibitory activity and selectivity have been investigated in a few studies. The experimental findings indicate that it can alter the intracellular redox status, lower intracellular lactate levels, alter the NADH/NAD + ratio, and kill tumor cells that lack mitochondrial respiration by blocking LDH (Hou et al., 2021). ATC cells often have increased LDH activity because they are strongly glycolytic-dependent. LDH inhibitors may therefore be more effective and sensitive in this subtype (Chen et al., 2021; Guo et al., 2024). However, it has also been proposed that FX11 lacks specificity, inhibits both LDHA and LDHB, and may cause certain negative effects (Zhao et al., 2022). It is also difficult to deliver FX11 to thyroid cancer tumor tissues. Thyroid cancer, particularly refractory ATC, may benefit from the use of LDH inhibitors, a novel anti-tumor medication that targets lactate metabolism. Although preclinical research has indicated some anti-tumor action for LDH inhibitors such GNE-140 and FX11, further study is necessary to verify their efficacy and safety in treating thyroid cancer as well as to look into potential combinations with other therapeutic medications.

### 5.3 Focusing on lactate transporters

Research on MCT1’s involvement in thyroid cancer is very limited. While some studies have demonstrated that MCT can be expressed in thyroid cancer cells, its precise role is unclear; it may be related to energy metabolism and the uptake and usage of lactate (Li et al., 2021; Felmlee et al., 2020). However, some research has shown that MCT4 expression is higher in thyroid cancer cells, particularly in undifferentiated thyroid cancer; MCT4 can also make the tumor microenvironment more acidic, encourage the buildup of lactate, and suppress immune cell activity (Silva et al., 2023; Khatami et al., 2019). Because ATC has high levels of MCT4 expression, MCT4 inhibitors may be effective in treating MCT4 and can be used in conjunction with immunotherapy, targeted therapy, or chemoradiotherapy to enhance the therapeutic benefit. One of the most extensively researched MCT inhibitors is α-cyano-4-hydroxycinnamic acid (CHC), which has been a hot topic in oncology therapy research and has shown anti-tumor promise in multiple preclinical trials (Duan et al., 2022). CHC is a derivative of cinnamic acid, with two key substituents (α-cyano and 4-hydroxy), which are very important for enhancing the activity of the compound and binding to MCT protein, α the synergistic effect of the two substituents of α-cyano and 4-hydroxy groups, significantly improving the binding ability of CHC to MCT protein and enhancing the inhibition of lactate transport (Pérez-Escuredo et al., 2016). The binding of CHC can cause conformational changes of MCT proteins, affect their transport functions, prevent monocarboxylic acid substrates such as lactate from binding to MCT proteins or prevent substrates from transmembrane transport, resulting in intracellular lactate accumulation, changing the acidity of the tumor microenvironment, causing tumor cells to lack energy, oxidative stress and metabolic disorders, and finally causing apoptosis (Kalyanaraman, Cheng, and Hardy, 2022; Payen et al., 2019). A variety of tumor cell lines, including glioblastoma, lung, breast, and colorectal, have shown anticancer activity in response to CHC, according to numerous *in vitro* studies (Combs et al., 2024; Chen J. et al., 2025; Ioannou et al., 2024). CHC’s poor bioavailability and short half-lives (Puri and Juvale 2020; Wu P. et al., 2021)further restrict its clinical application because it is not a selective inhibitor and may possibly have some effect on transporters other than MCT1 and MCT4, which could result in off-target effects and adverse effects. CHC is still a useful tool for researching MCT function and assessing treatment approaches that target MCT in spite of its drawbacks. Future studies must identify more selective, bioavailable, and less toxic MCT inhibitors and investigate their potential use in the treatment of thyroid cancer. CHC has prospective uses in thyroid research.

### 5.4 Combination therapy strategies

Because thyroid cancer is so varied, a single treatment might not be enough to stop the growth and spread of the tumor because tumor cells are highly adaptive and flexible and can develop a variety of resistance mechanisms in response to therapeutic stress (Haddadin and Sun, 2025). Additionally, tumor cells frequently change metabolic pathways to become resistant to drugs (Haddadin and Sun, 2025). For instance, BRAF-mutated melanoma cells might continue to produce via triggering glycolysis following BRAF inhibitor therapy (Huang and Kim, 2025). By altering the tumor cells’ microenvironment and metabolism, glycolysis inhibitors can have synergistic anti-tumor effects that increase the effectiveness of other therapeutic methods (Gu et al., 2025). Glycolysis inhibitors can limit glycolysis, lower intracellular ATP levels, change cell membrane permeability, and improve the absorption and distribution of chemotherapeutic drugs when used in conjunction with chemotherapy or targeted therapy (Niu et al., 2025; Li Z. et al., 2025). By preventing glycolysis, making tumor cells depend on other metabolic pathways, upsetting their metabolic adaption, and improving chemotherapy sensitivity and targeted therapy effectiveness, it can also prevent these compensatory processes (Khan et al., 2025; Wang Z. et al., 2025). Some preclinical and early clinical trials offer preliminary evidence, despite the fact that the clinical application of these inhibitors is still in its infancy and that there are comparatively few clinical studies on their use in conjunction when treating thyroid cancer with chemotherapy and targeted therapy. Research has demonstrated that the combination of LDH inhibitors and targeted medications (like BRAF inhibitors and MEK inhibitors) or metabolic inhibitors (like CHC) and chemotherapy medications (like doxorubicin and cisplatin) can prevent tumor growth, overcome tumor cell resistance to targeted therapy, and improve treatment efficacy (Jingtai et al., 2023; Aprile et al., 2023). For advanced or relapsed PTC and FTC, we can investigate the usage of lactate inhibitors in conjunction with RAI therapy using combination therapy approaches (Weis et al., 2025), lactate inhibitors in combination with BRAF inhibitors in patients with BRAF V600E mutations (Aprile et al., 2023; Huo et al., 2021), and lactate inhibitors in patients with severe disease who do not react to previous treatments, in addition to chemotherapeutic medicines. To evaluate the efficacy and safety of metabolic inhibitors in patients with advanced thyroid cancer when used in conjunction with targeted treatment and chemotherapy, future research must investigate the mechanism of combination application in greater detail, including the effects on cell signaling pathways, metabolic status, and the tumor microenvironment. Clinical trials must also be carried out.

## 6 Discussion

In addition to contributing to cell energy supply as the byproduct of glycolysis, lactate metabolism also affects tumor cell growth, metastasis, immune escape, and response to treatment by altering the tumor microenvironment. This makes lactate metabolism a complex and significant factor in the occurrence and progression of thyroid cancer. This review explains how lactate metabolism causes acidification and pro-angiogenesis, which change the tumor microenvironment, and elucidates the traits of thyroid cancer cells’ abnormal lactate metabolism. The mechanism of lactate metabolism in thyroid cancer can now be better understood because to our identification of the major signaling pathways that control lactate metabolism and the newly discovered lactylation alterations. Although some glycolysis and lactate transport inhibitors have advanced to the preclinical or clinical research stage, their effectiveness in treating thyroid cancer still requires additional validation. Targeted lactate metabolism has emerged as a new treatment approach for thyroid cancer. As a result, combination therapy might be a crucial area for further study. For example, the combination of metabolic inhibitors with chemotherapy and targeted therapy is predicted to reduce drug resistance in tumor cells and improve therapeutic efficacy. It is yet unknown how lactylation modification, an emerging post-translational alteration, specifically regulates metabolism, gene expression, and signaling pathway activation in thyroid cancer cells. The main proteins and enzymes involved in lactylation modification must be further identified in the future, and their implications on the phenotype of thyroid cancer must be clarified. However, since most current research is based on traditional cell culture models, more advanced *in vitro* models must be created in order to more accurately examine the role of lactate metabolism in thyroid cancer. To sum up, a thorough comprehension of the mechanism underlying lactate metabolism in thyroid cancer, the creation of more potent treatment approaches that target lactate metabolism, and personalized metabolic therapy will help to improve thyroid cancer metabolic therapy and provide patients fresh hope for a better outcome.
